# Case report: Transarterial chemoembolization for nasal hemangiosarcoma in a dog

**DOI:** 10.3389/fvets.2024.1505671

**Published:** 2025-01-21

**Authors:** Keunho Kim, Changgyu Lim, Songyi Kim, Hearang Lim, Sang-Hwan Hyun, Byeong-Teck Kang, Dongwoo Chang, Namsoon Lee

**Affiliations:** ^1^Section of Veterinary Medical Imaging, College of Veterinary Medicine, Chungbuk National University, Cheongju, Republic of Korea; ^2^Vet-ICT Convergence Education and Research Center (VICERC), Chungbuk National University, Cheongju, Republic of Korea; ^3^Laboratory of Veterinary Internal Medicine, College of Veterinary Medicine, Chungbuk National University, Cheongju, Republic of Korea

**Keywords:** dog, interventional radiology, nasal hemangiosarcoma, palliative treatment, TACE

## Abstract

This report outlines the use of transarterial chemoembolization (TACE) to treat a nasal tumor in a 10-year-old Welsh Corgi. The dog initially presented with persistent nasal discharge and facial deformity, which led to a diagnosis of nasal hemangiosarcoma. Although initial treatments with radiotherapy and chemotherapy provided temporary relief, the symptoms reappeared 11 months later. As a palliative measure, TACE was administered, utilizing carboplatin as the chemotherapeutic agent and gel foam as the embolic material. The procedure successfully reduced the tumor size and alleviated the dog’s symptoms. Follow-up CT scans at 1, 3, 7, and 10 months, demonstrated sustained tumor shrinkage. Interestingly, there was no reperfusion of the embolized vessels, indicating a prolonged therapeutic effect. No significant complications were observed during follow-up. This case highlights the potential of TACE as a palliative option for managing recurrent nasal tumors in dogs, especially when other treatments have limited efficacy. Further research is needed to establish TACE guidelines for treating nasal tumors in veterinary medicine.

## Introduction

Intranasal tumors represent about 1% of all canine tumors, with the majority being carcinomas such as adenocarcinomas, squamous cell carcinomas, and undifferentiated carcinomas (1). Typical symptoms of nasal tumors include respiratory issues, anemia from frequent epistaxis, nasal discharge and nasal congestion (1). Radiation therapy (RT) is the mainstay of treatment for nasal tumors, with most RT protocols requiring multiple treatment fractions, necessitating several anesthesia sessions (1). Surgical intervention may be limited due to a high risk of complications, such as severe intraoperative hemorrhage, subcutaneous emphysema, secondary rhinitis, and a reduced sense of smell post-surgery (1). In certain cases, surgery or RT may offer benefits, but these options are sometimes unfeasible due to tumor location, financial constraints, or lack of appropriate facilities (1). Given these challenges, interventional radiology techniques may provide an alternative for selected veterinary cancer patients. Procedures such as transarterial chemoembolization (TACE) may serve as palliative treatment options, aiming to reduce tumor size, slow progression, or manage symptoms like epistaxis, nasal congestion, and discharge (2, 3). TACE works by delivering chemotherapy directly to the tumor while obstructing its blood supply, leading to tumor shrinkage (4). However, research on the use of TACE for nasal cavity tumors remains limited.

This case report outlines the effective palliative treatment of a rare nasal hemangiosarcoma using TACE.

## Case description

**Figure 1:**
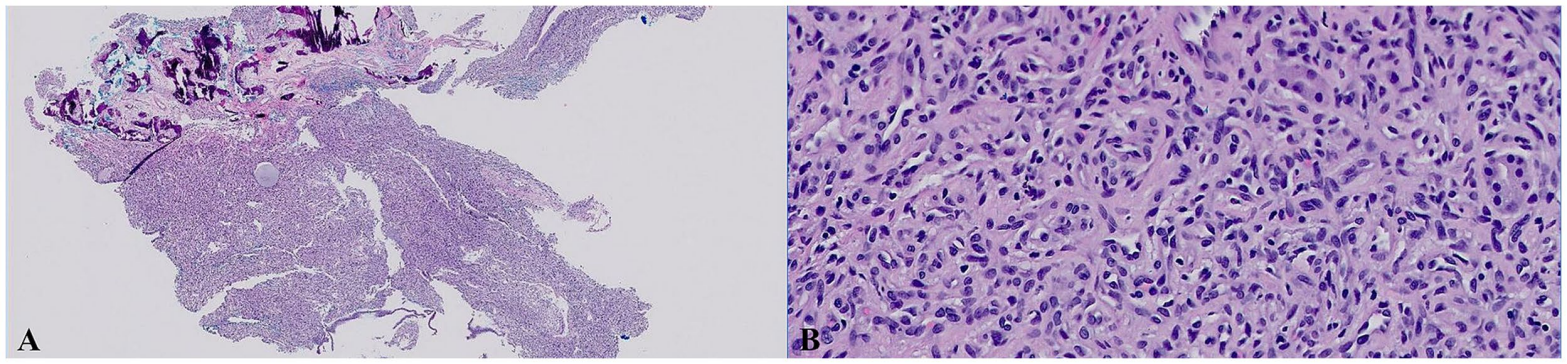
Histologic images of the samples of nasal mucosal tissue that were collected by endoscopic biopsies (H&E stain). **(A)** Low magnification (20×). **(B)** High magnification (400×). The section revealed an unencapsulated, modestly infiltrative vascular neoplasm extending through the submucosal stroma and to the specimen margins. The neoplasm featured polygonal to fusiform endothelial cells forming solid nests and incomplete vascular channels, supported by a collagenous stroma with capillary-like spaces and occasional larger cavities. These cells had a mitotic count of 4 (4 mitotic figures per 10 HPF), scant to modest eosinophilic cytoplasm, smooth polychromatic nuclei, and small indistinct nucleoli. Minimal secondary inflammation was noted, and no infectious agents were identified.

A 10-year-old castrated male Welsh Corgi weighing 13.5 kg, presented with nasal mucopurulent discharge persisting for over 6 months. Despite management with antibiotics and nonsteroidal anti-inflammatory drugs, the dog developed a facial deformity and nasal bleeding. Nasal endoscopy revealed a mass in the right nares, and samples were collected by endoscopic biopsy. Computed tomography (CT) scans and histopathology confirmed nasal hemangiosarcoma (Figure 1). Initial treatment with RT and adjuvant chemotherapy with doxorubicin temporarily improved respiratory symptoms and reduced the tumor size. However, the respiratory symptoms recurred after 11 months. Because we suspected chronic cardiac damage induced by doxorubicin, the chemotherapy regimen was changed to sorafenib, as indicated by the echocardiogram waveform changes.

A follow-up CT scan performed approximately 11 months post-RT show decreased nasal passage patency because of the increased size of the recurrent tumor and increased fluid accumulation in the frontal sinus. After consultation with the owner, it was decided to perform TACE as a palliative treatment.

**Figure 2:**
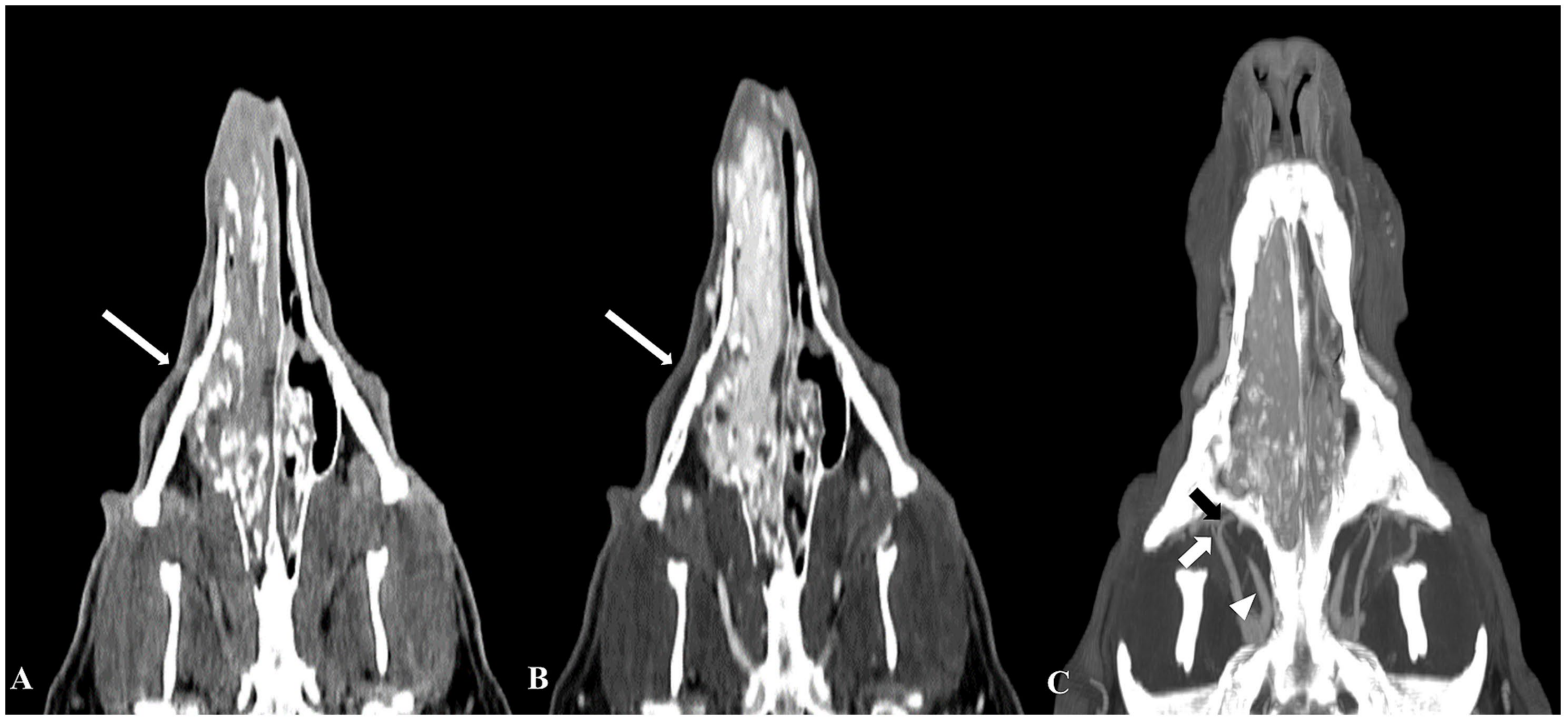
Computed tomography (CT) images of a nasal hemangiosarcoma and the tumor’s feeding arteries. Pre-contrast **(A)** and post-contrast **(B)** CT dorsal images revealed a diffuse mass extending from the right nostril to the mid-level of the nasopharynx. The mass showed significant hyperattenuation, indicating substantial vascularization **(B)**. The main feeding arteries to the mass were identified on a maximum intensity projection image **(C)**, including the right infraorbital artery (short white arrow), right external ophthalmic artery (white arrowhead), and right descending palatine artery (short black arrow).

Triple-phase CT scans using a 16-slice helical CT scanner (Aquilion Start, Canon Medical Systems, Otawara, Japan) were performed to obtain information on the feeding arteries to the mass. The CT protocol was as follows: 1.0-mm slice thickness, 120 kVp, 150 mAs, 512 × 512 matrix, and 0.75 s/rotation. Iohexol (Omnipaque 300; GE Healthcare, IL, United States) was used for intravenous contrast CT studies at a dose of 700 mgI/kg, administrated by a power injector (Salient CT Contrast Injector Single, IMAXEON Pty LTD, Pymble, Australia) with an injection rate of 2.5 mL/s. A bolus-tracking protocol was used during the arterial acquisition in a caudal to cranial direction, and triggered when the common carotid artery was reached at 80 HU after contrast injection, demonstrating the arterial phase. The venous phase scan was performed 10 s later, and a delayed phase scan was performed 2 min after the venous scan. On the CT, a diffuse mass lesion (approximately 10.1 cm × 3.4 cm × 4.5 cm in size) with calcification was observed in the right nasal cavity, appearing homogeneous and iso- to hypo-attenuated compared to the surrounding muscle tissue on pre-contrast images. The mass showed strong hyperattenuation in the arterial and venous phases, as indicated by significant vascularization. The mass extended into the left posterior nasal cavity, right eyelid, right olfactory bulb, and middle portion of the nasopharynx, obstructing the upper airway. The right frontal sinus showed fluid accumulation without contrast enhancement. In addition, severe erosive and permeative osteolysis was present in the surrounding bones, causing significant thinning and displacement of the nasal septum and vomer bone and loss of the bone margin at the right cribriform plate (Figures 2A,B). The tumor’s main feeding branches (infraorbital, descending palatine, and external ophthalmic arteries) were identified in the arterial phase (Figure 2C).

**Figure 3:**
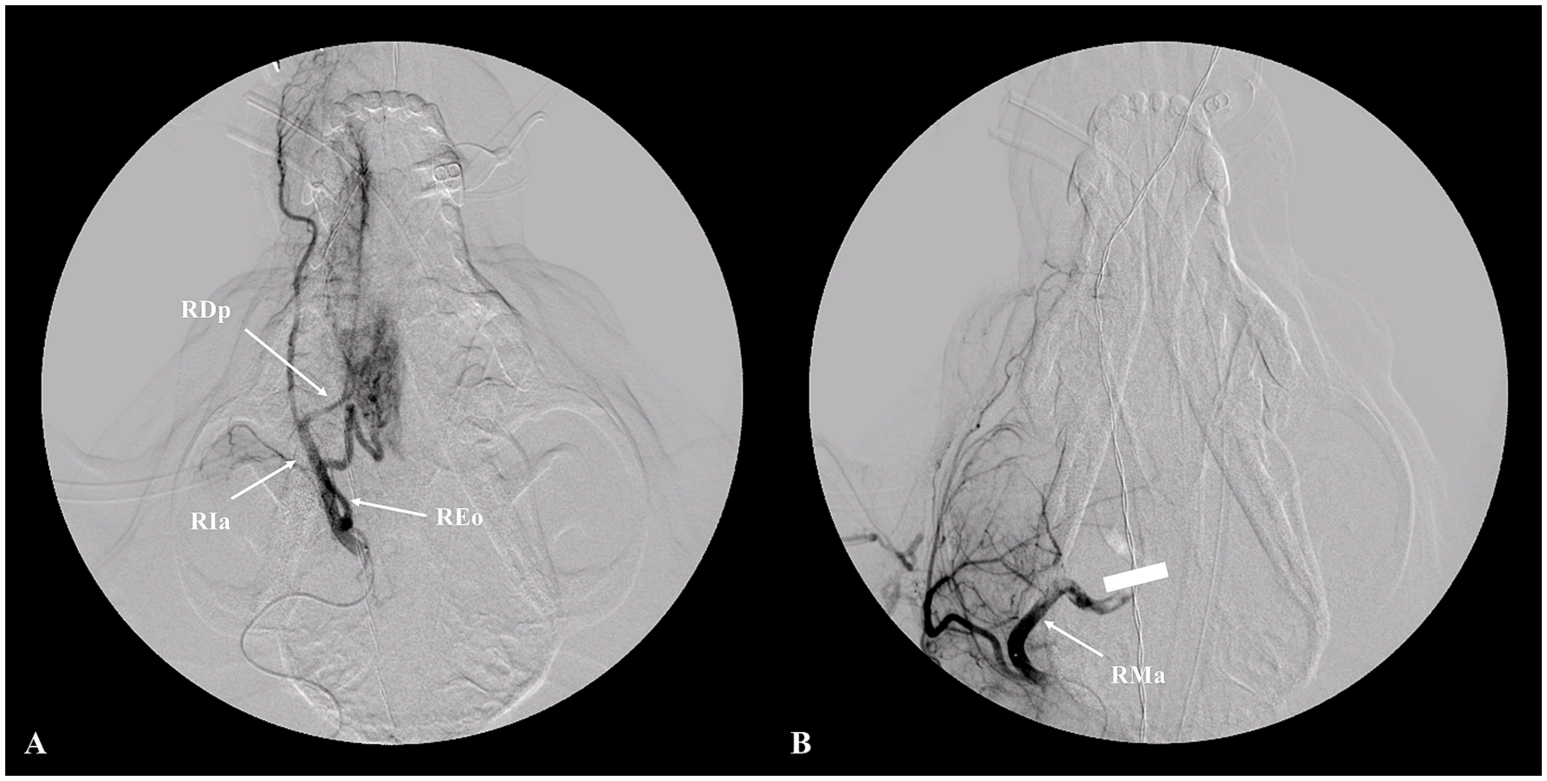
Angiographic imaging of the canine nasal hemangiosarcoma before and after embolization. Angiographic image of the canine head before embolization **(A)** showed the feeding arteries to the nasal mass (RDp, the right descending palatine artery; RIa, right infraorbital artery; REo, right external ophthalmic artery). The white bar indicates the embolization location; the feeding arteries were blocked and were not imaged **(B)**.

During the TACE procedure, the chemotherapeutic agent carboplatin was administered at a systemic dose (250–300 mg/m^2^ IV). Gel foam was selected as the embolic agent; a mixture of gel foam, 10 mL of carboplatin, and 10 mL of contrast agent was prepared. The procedure involves accessing the right femoral artery and advancing to the level of the external carotid artery. Angiography revealed the tumor had three major feeding branches, identified on the CT. Carboplatin was slowly injected into the caudal region of the external ophthalmic artery branch; fluoroscopy confirmed its entry into the tumor. The embolic agent mixture was administered slowly. After the procedure, angiography confirmed the blockage of the vessels supplying the tumor (Figure 3). Sorafenib, which had been used as a chemotherapy agent, was administered continuously before and after the TACE procedure.

**Figure 4:**
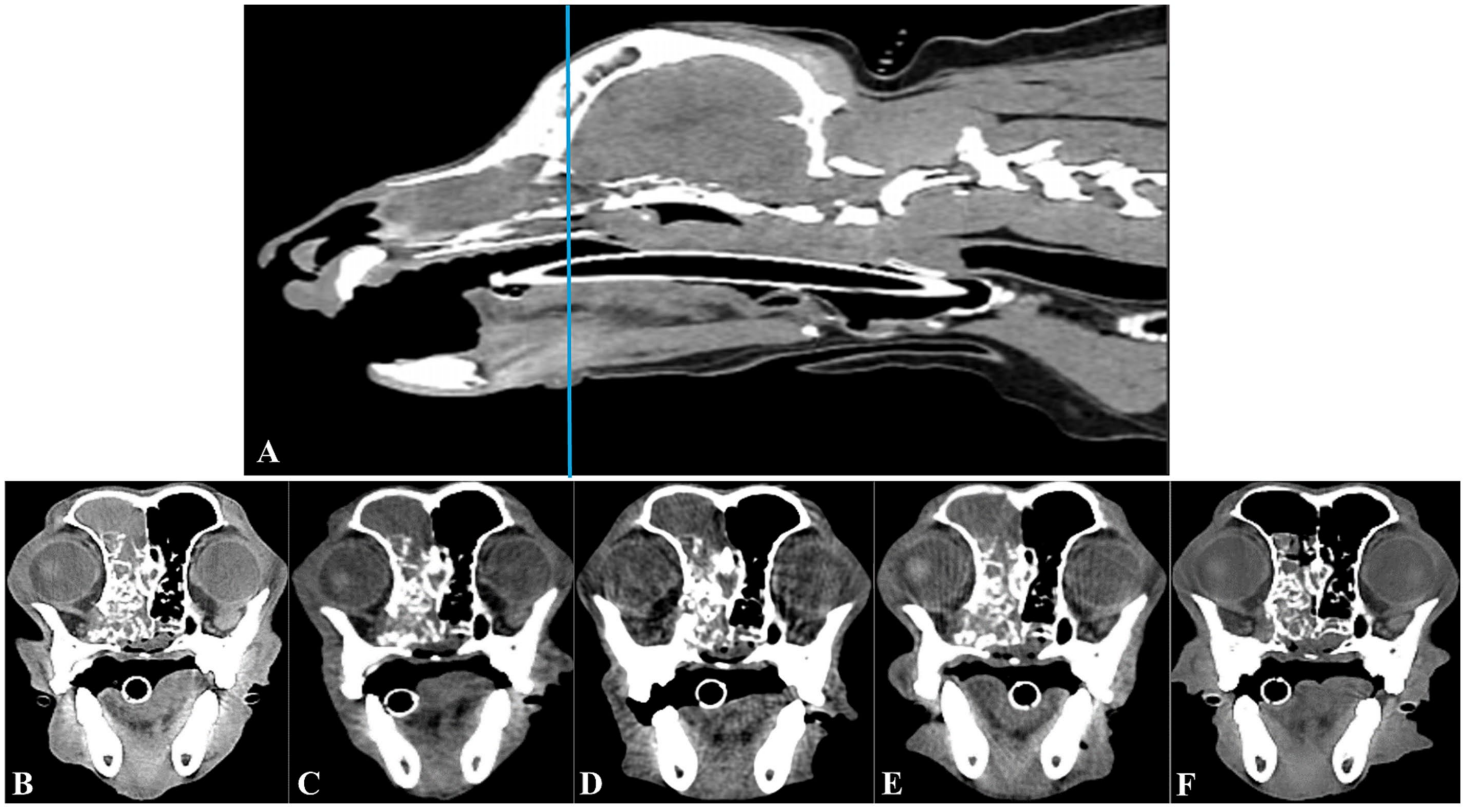
Longitudinal transverse CT imaging of the canine nasal hemangiosarcoma treated with transarterial chemoembolization (TACE). Sagittal CT imaging **(A)** shows the canine head; a horizontal line indicates the level corresponding to the transverse CT images presented in **B–F**. The transverse images provide a longitudinal view of the tumor’s response to TACE over time. Taken before the TACE procedure, the tumor’s initial size was clearly visible **(B)**. By 1 month after TACE, the tumor size was mildly smaller **(C)**. This reduction was sustained at 3 months, as seen in image **(D)**. At 7 months post-TACE, the tumor size remained reduced **(E)**, indicating ongoing control of the tumor. At 10 months after TACE, further tumor size reduction and improved fluid accumulation within the frontal sinus **(F)** were evident.

Symptoms (nasal bleeding and congestion) improved after TACE. Post-TACE follow-up CT scans at 1, 3, 7, and 10 months showed a sustained reduction in tumor size (Figure 4). Unexpected results were observed during continuous follow-up using CT. Despite expecting that reperfusion of the embolized vessel would occur within 4–6 weeks after the gel foam injection, as the embolic material was absorbed, post TACE follow-up observations at 10 months showed persistent occlusion with no evidence of blood reperfusion. The patient’s symptoms improved; continuous CT follow-up is ongoing.

## Discussion

Nasal tumors in dogs, especially hemangiosarcomas, are rare, highly vascular, and pose significant risks during surgical resection because of potential bleeding and morbidity (1, 5). In this case, CT imaging confirmed extensive vascularization within the tumor. RT is typically the preferred approach for managing canine intranasal tumors and is considered the standard of care (1). However, side effects such as alopecia, desquamation dermatitis, skin erythema, ocular discharge, and oral mucositis can negatively impact the patient’s quality of life (1, 3). Moreover, RT often necessitates multiple anesthesia sessions and may only provide temporary symptom relief, requiring further treatments. Another concern is that tumors that recur in previously irradiated areas can arise from radioresistant clonogenicity. Factors like intrinsic radio-resistance, hypoxia and the proliferation of previously irradiated cells contribute to the reduced efficacy of re-irradiation (6). The complexity of these factors largely depends on the underlying cause of recurrence. Therefore, in this case, we decided to perform TACE instead of an additional round of RT.

TACE could be a valuable palliative treatment option for nasal tumors, particularly when surgical intervention is either too risky or not feasible. By embolizing the blood vessels supplying the tumor, TACE can reduce or stop nasal bleeding and alleviate respiratory obstruction, thereby improving breathing and overall quality of life (7). In this case, high vascularity of the hemangiosarcoma likely contributed to the success of TACE, further supported by the use of sorafenib. This multi-kinase inhibitor works by inhibiting tumor cell proliferation and angiogenesis by targeting the vascular endothelial growth factor receptor (8, 9). As it combines localized delivery of chemotherapy with embolization to induce immediate tumor ischemia and shrinkage, TACE can provide faster symptom relief compared to RT, which typically takes about 3 weeks to respond; it can vary depending on the size of the tumor (10). Although there is limited veterinary research on TACE for nasal tumors and no standardized treatment protocols exist, this case effectively highlights the treatment’s potential efficacy in such situations.

While TACE does come with certain risks, such as non-target embolization and pain, and potential complications like necrosis or pathological fractures (11), none of these issues were encountered in this case. The use of advanced imaging technologies, such as triple-phase CT scans and fluoroscopy, plays a vital role in minimizing risks and ensuring accurate delivery of the embolic material (12). Triple-phase CT is particularly useful for precisely identifying the tumor’s location, size, and blood supply, helping guide the catheter directly to the tumor and enabling the efficient administration of chemotherapy and embolic agents while minimizing damage to nearby tissues (12). This method is highly effective in visualizing the feeding arteries during the arterial phase; knowing their exact location in advance reduces the need for catheter repositioning during the procedure, resulting in shorter anesthesia duration and less physical strain on the patient, which in turn promotes faster recovery (12). Such careful planning likely contributed to a shorter hospital stay and a quicker return to normal activities.

Unexpectedly, a follow-up CT scan performed 10 months after TACE revealed persistent vascular occlusion without any signs of blood reperfusion. Although the exact reason for this prolonged vascular embolization remains unclear, several possibilities could explain this outcome. One potential cause is the immune system’s reaction to the embolic material, which could trigger inflammation and fibrosis, thereby extending the duration of the occlusion (13). Another possibility is that mechanical damage to the vessel wall during embolization might disrupt the endothelium, resulting in scar formation and contributing to the persistence of the occlusion. Since no similar cases have been documented, pinpointing a definitive cause for this phenomenon is challenging. Due to the absence of well-established protocols and the limited amount of research on TACE for nasal tumors in veterinary medicine, further studies with larger sample sizes are necessary to standardize treatment protocols and develop clear guidelines. Additional research is also required to evaluate the long-term outcomes of this treatment.

In conclusion, while nasal tumors in dogs generally respond well to RT, patients with recurrent or worsening symptoms may benefit from TACE, especially in cases of highly vascularized tumors. Further research is necessary to develop standardized protocols for the use of TACE in the treatment of canine nasal tumors.

## Data Availability

The original contributions presented in the study are included in the article/supplementary material, further inquiries can be directed to the corresponding authors.
